# Oral Antioxidant Treatment of Men Significantly Improves the Reproductive Outcome of IVF Cycles

**DOI:** 10.3390/jcm10153254

**Published:** 2021-07-23

**Authors:** Paola Scaruffi, Emanuele Licata, Elena Maccarini, Claudia Massarotti, Francesca Bovis, Fausta Sozzi, Sara Stigliani, Alessandro Dal Lago, Ida Casciano, Rocco Rago, Paola Anserini

**Affiliations:** 1 UOS Physiopathology of Human Reproduction, IRCCS Ospedale Policlinico San Martino, 16132 Genova, Italy; elena.maccarini@hsanmartino.it (E.M.); fausta.sozzi@hsanmartino.it (F.S.); sara.stigliani@hsanmartino.it (S.S.); ida.casciano@hsanmartino.it (I.C.); paola.anserini@hsanmartino.it (P.A.); 2 UOSD Physiopathology of Reproduction and Andrology Unit, Ospedale Sandro Pertini—ASL Roma 2, 00159 Rome, Italy; emanuele.licata@aslroma2.it (E.L.); Alessandro.dallago@aslroma2.it (A.D.L.); rocco.rago@aslroma2.it (R.R.); 3 Department of Neurosciences, Rehabilitation, Ophthalmology, Genetics, Maternal and Child Health (DINOGMI), Academic Unit of Obstetrics and Gynecology, University of Genova, 16132 Genova, Italy; claudia.massarotti@unige.it; 4 Department of Health Sciences (DISSAL), University of Genova, 16132 Genova, Italy; francesca.bovis@unige.it

**Keywords:** male fertility, oral antioxidants, myo-inositol, alpha-lipoic acid, sperm quality, ICSI, IVF outcomes

## Abstract

Some 30% to 80% of male sub-fertility may be associated with oxidative stress that damages spermatozoa and can decrease success of in vitro fertilization (IVF) techniques. This multicenter, longitudinal, prospective study aimed to investigate whether oral antioxidant supplementation improved the reproductive competence of men who had had low fertilization rates in their previous intracytoplasmic sperm injection (ICSI) cycles without azoospermia or severe oligozoospermia or any identifiable andrological disease. Seventy-seven men from couples who had an ICSI attempt with unexplained <60% fertilization rate took an antioxidant mix of myo-inositol, alpha-lipoic acid, folic acid, coenzyme Q10, zinc, selenium, and vitamins B2, B6, and B12. Semen parameters were analyzed before (T0) and after 90 days (T90) of treatment, and outcomes of the paired T0 and T90 cycles were compared. After the treatment there was an increase in sperm concentration (*p* = 0.027), total motile sperm count (*p* = 0.003), progressive motility (*p* < 0.0001), and a decreasing trend of DNA-fragmented spermatozoa. Embryological outcomes (fertilization, embryo quality, blastocyst development) were significantly higher in T90 than T0 cycles. No T0 cycle resulted in an evolutive pregnancy. Conversely, in T90 cycles 29 singleton clinical pregnancies were obtained. No negative neonatal outcomes were recorded in newborns after antioxidant treatment. Diet supplementation of men who have had low fertilization rates in their previous ICSI cycles with a combination of myo-inositol, alpha-lipoic acid, folic acid, coenzyme Q10, zinc, selenium, betaine, and vitamins may improve semen reproductive potential and ICSI clinical outcome.

## 1. Introduction

Human infertility affects 15% of couples of childbearing age, of which 30–40% is due to male infertility. Alterations in sperm number, motility, and morphology may be related to various factors, i.e., varicocele, cryptorchidism, hypogonadism, infections, traumas, tumors, smoke, or exposure to environmental agents. In fact, such conditions are associated with decreased fertility (e.g., up to 10% of patients with unilateral undescended testis will develop infertility, and varicoceles occur in about 40% of infertile males) because they burden testicular function, negatively affecting sperm quality and spermatogenesis overall. Specifically, cryptorchidism impairs Sertoli cell function and possibly Leydig cell function as well. Varicoceles leads to increased oxidative stress within the testicular parenchyma which is thought to be caused by scrotal hyperthermia, testicular hypoxia, and blood-testis barrier disruption. Nevertheless, 25–30% of infertile patients’ etiology is not clear [1], and between 30% to 80% of male sub-fertility may be associated with oxidative stress (OS) [2].

Reactive oxygen species (ROS) are highly reactive oxidizing agents that physiologically have positive effects on sperm function: for instance, hydrogen peroxide stimulates the acrosome reaction and sperm hyperactivation, ultimately boosting the sperm’s transit through cumulus and binding to the zona pellucida ZP-3 protein [3]. In semen there is homeostasis between free radicals, mainly produced by leukocytes and spermatozoa, and protective enzymatic (superoxide dismutase, catalase, glutathione peroxidase) and non-enzymatic (ascorbic acid, α-tocopherol, glutathione, albumin, carnitine, carotenoids, flavonoids, urate, prostasomes) antioxidants. Some clinical conditions (varicocele, prostatitis, autoimmunity), environmental factors (high temperatures, electromagnetic radiation, pesticides, polluting agents), lifestyles (cigarette smoke, alcohol abuse, drug addiction), and nutritional errors (hyperlipidic, iperproteic diet, obesity, poor diet) may alter this equilibrium state and lead to a harmful accumulation of ROS in seminal fluid [4]. Spermatozoa are particularly vulnerable to oxidative damage because their plasma membranes are rich in polyunsaturated fatty acids and have low concentrations of scavenging enzymes. Such a ROS-induced peroxidation of the sperm membrane causes a decrease in its flexibility, and therefore of tail motion. Sperm motility may also be impeded by direct ROS damage to mitochondria. Moreover, free radicals damage sperm DNA by attacking the purine and pyrimidine bases and the deoxyribose backbone or by leading to apoptotic caspase-mediated enzymatic DNA degradation [5].

A relationship between OS and poor clinical outcome of in vitro fertilization (IVF) procedures has been observed. ROS in seminal plasma were found to have a negative correlation with fertilization rate, embryo development, and pregnancy rate [6].

Oral supplementation with antioxidants may improve sperm quality by reducing OS [7], thus dietary supplementation with antioxidants has gained much attention in recent years, although evidence for the optimal antioxidant regimen that can be efficient in clinical practice is uncertain [8].

To date, few studies have looked into the effect of antioxidant oral supplements on clinical outcome of IVF cycles [9,10,11,12,13,14] and a clear consensus about the effectiveness of antioxidant therapy is still lacking [7,8]. This is principally because studies investigating different antioxidant forms vary considerably in the dosage and combinations used, as well as outcome measures.

We previously reported that a mixture of nutraceuticals (myo-inositol, alpha-lipoic acid, folic acid, betaine, and vitamins) significantly improved sperm parameters in sub-fertile men [15].

We aimed at assessing the effects of such an oral antioxidant supplementation of men who had had low fertilization rates in their previous ICSI (intracytoplasmic sperm injection) cycles by evaluating embryological, clinical, and neonatal outcomes.

## 2. Materials and Methods

### 2.1. Study Design and Participants

**Figure 1:**
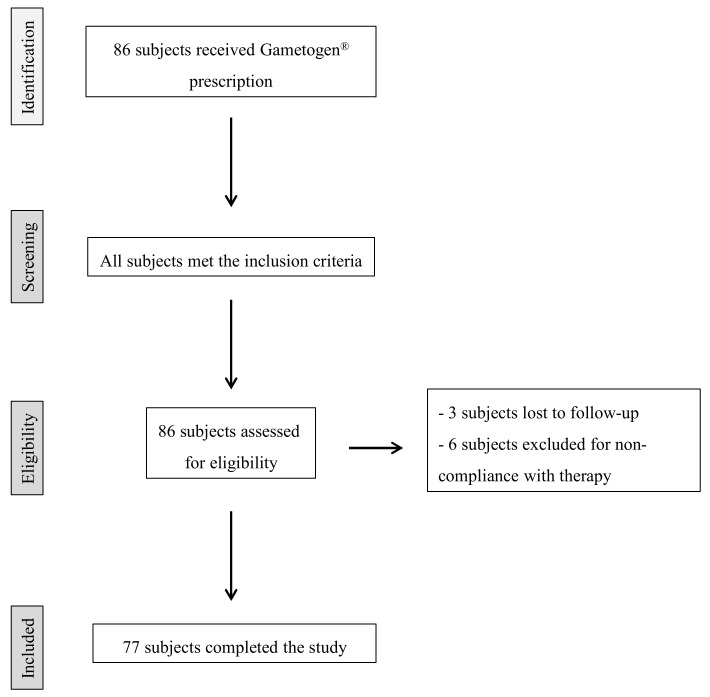
Flowchart of patient recruitment.

This multicenter, longitudinal, prospective study was performed in 2018–2020 at UOS Physiopathology of Human Reproduction, IRCCS Ospedale Policlinico San Martino, Genova and at UOSD Physiopathology of Reproduction and Andrology Unit, Ospedale Sandro Pertini—ASL Roma 2, Roma. The study design is outlined in Figure 1.

We recruited 86 couples who had a history of a homolog ICSI attempt using fresh oocytes and ejaculated sperm with unexplained <60% fertilization rate, inseminated oocytes >6, and female partner <38 years old. Male inclusion criteria: sperm count > 1 × 10^6^/mL and no concomitant consumption of drugs. Patients with azoospermia or severe oligozoospermia (sperm count less than 1 × 10^6^/mL) or with an identifiable cause of infertility (leukocytospermia and/or positive sperm culture, epididymo-orchitis, prostatitis, inguinoscrotal surgery, cryptorchidism, varicocele, etc.) were excluded. Female partners with diminished ovarian reserve (less than six retrieved oocytes in a prior IVF cycle or elevated early follicular phase FSH (follicle-stimulating hormone) or less than 1 ng/mL Anti-Müllerian Hormone concentration in serum) were excluded from the study. We excluded any type of endocrine, metabolic, autoimmune, and neoplastic diseases in both female and male partners.

The 86 male partners fulfilled the inclusion criteria and began Gametogen^®^ treatment (Laborest S.r.l., Nerviano, Italy). Two Gametogen^®^ tablets contained myo-inositol (1000 mg), alpha-lipoic acid (800 mg), folic acid (400 mg), coenzyme Q10 (200 mg), zinc (15 mg), selenium (83 µg), and vitamins B2 (2.8 mg), B6 (2.8 mg), and B12 (5 µg). Nine subjects did not complete the study: three were lost to follow-up and six were excluded for non-compliance with therapy. Therefore, a total of 77 subjects/couples completed the study and were included in statistical analyses. No side effects due to the oral administration of Gametogen^®^ were observed in any participants.

Information on demographics, etiology of infertility, male partner’s BMI, and smoking habits of the 77 couples were collected, as outlined in Table 1. Female infertility was caused by tubaric factor in 10% of cases, ovulatory dysfunction in 9%, and endometriosis in 4%.

Male partners had one or more altered semen parameters according to WHO 2010 criteria [16] in 74% (57/77) of cases. Specifically, at baseline 4% of them presented oligospermia, 19% asthenospermia, and 51% both oligospermia and asthenospermia. Moreover, patients had a body mass index (BMI) of between 19.8 kg/m^2^ and 35.9 kg/m^2^ (average BMI: 25.5 ± 3.1 kg/m^2^), and 34% (26/77) were smokers (maximum < 20 cigarettes/day).

After 90 days of the antioxidant treatment, each couple performed a second ICSI cycle. Semen parameters were analyzed at baseline (T0) and after 90 days (T90) of treatment, and embryological, clinical, and neonatal outcomes of the paired T0 and T90 cycles were compared.

### 2.2. Outcomes Measures

The primary outcome was the clinical pregnancy rate (defined as pregnancies with at least one gestational sac divided by number of embryo transfers). The secondary outcomes were fertilization rate (defined as the ratio between the number of fertilized oocytes and the number of MII oocytes injected/inseminated), cleavage rate (defined as the ratio between the number of cleaved embryos and the number of fertilized oocytes), quality of embryos, blastocyst development rate (defined as the ratio between total number of blastocysts formed and the number of embryos cultured up to day 5–7), implantation rate (defined as fetal cardiac activities at 12 weeks of gestation divided by number of transferred embryos), live-birth rate (defined as live-born babies divided by number of transferred embryos), cumulative clinical pregnancy rate per couple (defined as pregnancies with at least one gestational sac—after ET (embryo transfer) of either fresh or frozen embryos—divided by number of couples), cumulative live-birth rate per couple (defined as live-born babies—either after ET of fresh or frozen embryos—divided by number of couples), and any adverse event including miscarriage rate (defined as abortions divided by number of pregnancies). We also evaluated the outcome of the newborns by collecting their birthweights, expressed as percentile and standard deviation score (SDS) for gestational age according to the Italian reference curves [17].

### 2.3. Sperm Analysis

At T0 and T90, semen samples were obtained after three days of sexual abstinence. They were collected into sterile containers and held at room temperature for 30 min to liquefy. After liquefaction, all sperm samples were processed through the two-layer density gradient method (Sydney IVF Sperm Gradient, Cook Medical, Sydney, Australia) according to the manufacturer’s instructions. Basic sperm analysis was carried out according to the WHO (World Health Organization) criteria [16], including sperm count, concentration, and motility. Sperm score for motility evaluation was expressed as grades a to d, and progressive motility rate was calculated as the percentage of a + b.

### 2.4. Evaluation of Sperm DNA Fragmentation

Visualization of fragmented sperm nuclear DNA was performed through SCD (Sperm Chromatin Dispersion test) by using Halo-sperm^®^ kit (Halotech DNA SL, Madrid, Spain), according to the manufacturer’s instructions. Briefly, a pre-provided microcentrifuge tube containing 1% low-melting-point agarose was placed in boiling water for 5 min and then transferred to a 37 °C water bath for equilibration. Immediately after, 50 µL of fresh semen sample previously diluted in PBS to a maximum of 20 million sperm/µL was added to the melted agarose tube. A drop of 10 µL of this agarose-semen sample was pipetted onto a pre-treated slide and covered with a coverslip and the slide was then incubated at 4 °C for 5 min to solidify the agarose. The slide was incubated for 7 min in a provided acid denaturant. Afterwards, the slide was incubated in 10 mL of lysis solution at room temperature for 25 min. The slide was washed with abundant distilled water to completely remove the lysis solution and then sequentially dehydrated using 70%, 90%, and 100% ethanol (Sigma Aldrich, St. Louis, MI, USA) for 2 min each. The slides were air-dried and stained using Brightfield Staining Kit (Halotech DNA SL): the slide was incubated in Staining Solution A (fluored^®^) for 6 min and in Staining Solution B (fluogreen^®^) for 7 min. Then, the slide was examined under ×400 magnification using a phase contrast microscope (Eclipse E200, Nikon, Tokyo, Japan). Each slide was scored for minimum 300 sperm spermatozoa following these criteria: (i) spermatozoa without DNA fragmentation: spermatozoa with big- or medium-sized halo; (ii) spermatozoa with fragmented DNA: spermatozoa with small halo or without halo or with a core irregularly or weakly stained (degraded). The Sperm DNA Fragmentation (%) was calculated as ((fragmented + degraded sperm)/total sperm counted) × 100.

### 2.5. Assisted Reproduction Techniques

Controlled ovarian hyperstimulation, oocyte recovery, ICSI technique, embryo culture, and morphological assessment of embryos were performed as described previously [18]. Embryo transfer (ET) was generally performed 72 h after oocyte collection. However, if the patient had only one to two fertilized oocytes, it could have been carried out at day 2, and if she had at least four good quality embryos on day 3, day 5 transfer at the blastocyst stage was considered. Surplus embryos that developed up to blastocyst stage were cryopreserved by vitrification [19].

### 2.6. Vitrification and Warming of Blastocysts

Blastocysts were vitrified using a commercial kit (Vitrification Freeze Kit, Irvine Scientific, Santa Ana, CA, USA). Briefly, the blastocysts were equilibrated at room temperature for 12–15 min in an equilibration solution (ES) containing 7.5% ethylene-glycol (EG) and 7.5% dimethyl-sulfoxide (DMSO) and then transferred in a vitrification solution (VS) with 15% EG, 15% DMSO, and 0.5% sucrose for 1 min. During this phase, the blastocysts were rinsed to dilute the excess of ES. In the last 10 s, the blastocysts were placed onto the tip of a Cryotop (Kitazato, Tokyo, Japan) surrounded by a minimal volume of VS. The devices were lastly plunged into liquid nitrogen. For the warming procedure, the Cryotop was immediately moved from liquid nitrogen into a thawing solution of 1 M sucrose for 1 min at 37 °C. Then, the blastocysts were moved to a dilution solution of 0.5 M sucrose for 3 min and lastly into a washing solution for 5 min at room temperature. The blastocysts were then cultured in Sydney IVF Blastocyst medium (Cook Medical, Bloomington, IN, USA) at 37 °C for at least 2 h before ET.

### 2.7. Data Collection and Statistical Analyses

Other than general patient demographic and clinical data, we retrieved semen parameters and embryological and clinical outcomes for each cycle. Descriptive statistics were reported as mean ± standard deviation (SD) for continuous variables and as frequencies and percentages for categorical variables. Comparisons of data before and after antioxidant treatment was performed using chi-square test or Wilcoxon signed ranks test as appropriate. A *p*-value < 0.05 was considered statistically significant when comparing clinical pregnancy rates before and after treatment. Adjustment for multiple comparisons was performed when assessing secondary endpoints. To control the rate of the overall type I error, the Hochberg stepwise test was performed.

Analyses were carried out using MedCalc^®^ 11.5.1.0 software (MedCalc Software Ltd., Mariakerke, Belgium) and SAS 9.4 (SAS Institute, Cary, NC, USA).

## 3. Results

### 3.1. Semen Parameters before and after Oral Antioxidant Treatment

Sperm parameters of the participants at baseline (T0) and after 90 days (T90) are presented in Table 2.

Semen quality improved after 90 days of treatment, with a statistically significant increase in sperm concentration (*p* = 0.027), number of spermatozoa (*p* = 0.040), progressive motility (*p* < 0.0001), and total motile sperm count (*p* = 0.003) with respect to baseline.

After therapy with antioxidants, a decreasing trend was observed (−42%) of DNA-fragmented spermatozoa, which did not reach a statistically significant level probably due to the small number of paired data available. Unfortunately, direct measure of OS were not available.

### 3.2. Embryological Outcomes of Cycles with Ejaculated Spermatozoa before and after Oral Antioxidant Treatment

Characteristics of the cycles are reported in Table 3. There was no significant difference for female age between the two sequential ICSI attempts performed before and after the oral antioxidant treatment. After the antioxidant treatment, the number of mature oocytes retrieved was not statistically different in comparison to previous attempts, but with the same number of injected oocytes the fertilization rate showed a statistically significant increase (*p* < 0.001). Regarding embryos, there was a significantly higher number of cleavage stage embryos (*p* < 0.0001) with good morphology (*p* < 0.0001) in the T90 cycles. The proportion of patients who developed at least one blastocyst in the cycle after the antioxidant treatment was significantly higher (*p* < 0.0001) with respect to baseline, with a significantly higher number of blastocysts developed (*p* = 0.001) with respect to T0 attempts.

### 3.3. Clinical Outcomes of Cycles with Ejaculated Spermatozoa before and after Oral Antioxidant Treatment

In the T0 cycles, nine patients had no embryos to transfer, one ET was postponed because of high serum progesterone level, and another one because patient anemia occurred after oocyte retrieval. In T90, one ET was postponed because of high serum progesterone level and two others because of ovarian hyperstimulation (Table 4).

The higher number of cleavage stage embryos with good-morphology after the antioxidant treatment preferentially determined the choice of day 5 ET (*p* < 0.0001) in the T90 cycles with respect to baseline attempts. Overall, only two clinical pregnancies, both of which were singletons and ended in spontaneous abortions, were obtained in the attempts performed before the antioxidant treatment, whereas the cycles performed after the oral antioxidant treatment resulted in 25 successful singleton clinical pregnancies. Consequently, significant differences in the clinical pregnancy (3% (2/68) versus 34% (25/74), *p* < 0.0001) and implantation (2% (2/128) vs. 21% (25/117), *p* < 0.0001) rates were observed between the two sequential attempts. Moreover, during the study period, 12 patients received 13 vitrified-warmed ET, which included four and nine blastocysts from T0 and T90 cycles, respectively. Altogether, cycles performed after the antioxidant treatment had significantly higher cumulative clinical pregnancy and live birth rates (*p* < 0.0001) with respect to baseline cycles (Table 4).

### 3.4. Perinatal Characteristics of Babies from Cycles with Ejaculated Spermatozoa after Oral Antioxidant Treatment

A total of 22 neonatal outcomes from T90 cycles were available, all of them from singleton gestations. Only for one newborn were perinatal data lost at follow-up. As detailed in Table 5, all babies were classified as appropriate for gestational age on the basis of the centiles. There were not preterm babies. No stillbirths and no malformations were recorded.

## 4. Discussion

Fertilization failure in IVF treatments has mainly been attributed to oocyte activation deficiencies that can be caused by both oocyte (i.e., defects in proteins involved in the fertilization process, cytoplasmic immaturity and spindle abnormalities, and low oocyte competence associated with poor ovarian response) and sperm (i.e., failed sperm head decondensation, premature chromatin condensation, sperm aster defects, and altered sperm motility and progression) related factors [20]. In this context, high levels of ROS, which underlie mis-balanced antioxidant–oxidant molecules, have been proven to be one of the most damaging factors that suppresses sperm capacitation and acrosome reaction, two pivotal events for normal sperm-oocyte fertilization. In a previous study, we proved that combined treatment with myo-inositol, alpha-lipoic acid, folic acid, betaine, and vitamins of sub-fertile men significantly improved their sperm parameters [15]. Hereby, we demonstrated that such an antioxidant diet supplementation improved semen reproductive potential of men whose sperms, although without severe abnormalities, were previously unable to optimally activate the oocytes. This study is the first report describing the positive impact of the aforementioned antioxidant male diet supplementation on embryological and clinical outcomes of IVF procedures.

Since OS negatively impacts the sperm membrane, and hence the sperm’s physiological processes, some authors claim that ROS were negatively associated with fertilization only in conventional IVF and that only conventional IVF could benefit from antioxidants [10,13,21]. Intriguingly, our data demonstrated that ICSI, although it bypasses conventional IVF-related sperm processes (i.e., the sperm hyperactivation, the membrane fusion and the acrosome reaction), also benefits from oral antioxidants’ effects, in accordance with some available studies [9,11,12,14]. In this regard, impaired sperm chromatin compaction may contribute to fertilization failure after ICSI, since it may induce DNA fragmentation and the proliferation of free radicals, which can break the DNA backbone indirectly by reducing protamination and disulphide bond formation [22]. The link between DNA breaks and ROS has been recently established and there is a growing trend of antioxidant supplementation to counteract sperm DNA fragmentation [8]. In accordance, our results on sperm DNA fragmentation, although preliminary and with a small number of paired data, showed a decreasing trend in DNA-fragmented spermatozoa after therapy with antioxidants. Thus, the observed increase in fertilization rates in T90 ICSI may be a consequence of an ameliorated DNA integrity thanks to an efficacious reduction in oxidative attacks.

Moreover, since our sperm DNA fragmentation data are currently not conclusive and the significantly improved semen parameters after treatment (i.e., concentration, motility) do not directly impact on ICSI, we speculate that out findings possibly reflect better testicular health in general.

The improved sperm competence after antioxidant supplementation was also demonstrated in this study by the significantly better embryo quality in the cycles performed at T90, in terms of higher proportion of good-morphology embryos with respect to baseline cycles. Moreover, the proportion of patients who developed at least one blastocyst in the cycle after the antioxidant treatment was significantly higher with respect to the baseline, with a significantly higher number of blastocysts developed with respect to T0 attempts.

Previous studies reported that unrepaired DNA damage was responsible for the so-called “late paternal effect” [23], which has adverse effects on embryo development to the blastocyst stage and viable pregnancy rate [10,13,21]. The late paternal effect occurs because embryo development is dependent on maternal factors at the time of fertilization until day three, when the paternal genome begins to be transcribed [24]. However, recent findings have shown that reactive oxygen species in semen also influence the early stages of embryo development [25], which is consistent with the observations reported in the present study. In fact, sperm ROS-induced DNA damage might be translated into chromosome aberrations after the first metaphase and affect a wide variety of cellular processes, including DNA repair mechanisms, transcription, and cell cycle control [26]. Thus, if the level of DNA damage exceeds the oocyte’s capacity to completely repair the damage, then the cells might undergo apoptosis and directly reduce the embryo cleavage rate.

Despite the aforementioned embryological data showing the effectiveness of the antioxidant supplementation taken by males, we chose pregnancy as the primary endpoint, since we believe it is a better marker of improved fertility potential. A recent analysis of the literature of the last ten years concerning the effects of low molecular weight antioxidants (ascorbic acid, N-acetylcysteine, alpha-tocopherol, and CoQ10, administered as a single antioxidant or as a combination of different antioxidants plus vitamins and/or micronutrients) showed that only ten studies included pregnancy and/or live birth rates as an outcome measure to determine the effects of the therapy [27]. Unfortunately, only two reports found improvement of the pregnancy rate in the group of antioxidant-treated patients in scientifically rigorous case–control studies [28,29]. Furthermore, very few studies evaluated pregnancy rates after antioxidant therapy in IVF treatments [10,11,12,13,14]. Although they found a positive effect, the clinical heterogeneity of the results, the different drugs used, and the different doses administered, together with a paucity of prospective randomized controlled trials and different inclusion criteria, contributed to a lack of conclusive evidence. Therefore, the role of antioxidants in boosting pregnancy rates is still a matter of debate. In consideration of the above, the primary outcomes of this study was pregnancy rate. The results of this study demonstrate that the treatment of men with an antioxidant combination of nutraceuticals positively affected the reproductive capacity of infertile couples in terms of higher pregnancy and live birth rates.

We are aware that some limitations should be considered: firstly, the study sample was small and results need to be confirmed in a larger sample size to correct primary endpoints for putative confounders; secondly, it was a longitudinal, prospective study and a randomized controlled trial would confirm its clinical value; thirdly, we used a mixture of different oral nutraceuticals, thus we cannot infer whether the observed results could be due to the action of a single component or to their mixture. Last but not least, we would like to deeply investigate the direct effect of the antioxidant treatment on semen oxidative status.

Our data confirmed the safety of myo-inositol, alpha-lipoic acid, folic acid, coenzyme Q10, zinc, selenium, and vitamins B2, B6, and B12 not only because no side effects due to the oral administration of Gametogen^®^ were observed in any participants, but also because neither malformations nor negative neonatal outcomes were recorded in newborns after the antioxidant treatment. To the best of our knowledge, in literature no reports are available about perinatal characteristics of newborns after fathers’ antioxidant diet supplementation. Thus, we believe that the strength of this study is that it showed for the first time that the treatment of men with an antioxidant combination of nutraceuticals markedly improved the chance of successful healthy childbirth during ICSI treatments. Actually, it is important to remember that helping infertile couples to conceive a healthy baby is the goal of assisted reproductive treatments and this study, despite its aforementioned limitations, showed that such an antioxidant approach may be a valid method to increase the chances of becoming pregnant for infertile couples with a previous unexplained failed ICSI attempt.

## 5. Conclusions

The findings of this study indicate that antioxidant treatment of men, who had had low fertilization rates in their previous ICSI cycles, may have a beneficial effect on sperm reproductive potential. Therefore, such a nutraceutical approach represents a valid method for improving ICSI clinical outcomes.

## Figures and Tables

**Table 1:** Baseline demographic and fertility characteristics of the 77 couples included in the study.

Female age (years; mean ± SD, range)	35.3 ± 3.2, 20–38
Male age (years; mean ± SD, range)	38.6 ± 4.8, 30–53
Etiology of infertility (*N*, %)	
Idiopathic	31/77, 40%
Female	10/77, 13%
Male	26/77, 34%
Combined	10/77, 13%
Male BMI (kg/m^2^, mean ± SD, range)	25.5 ± 3.1, 19.8–35.9
Male smoking habit (*N*, %)	26/77, 34%

SD, standard deviation; BMI, body max index.

**Table 2:** Sperm parameters assessed in 77 patients before and after treatment.

	T0	T90	*p*-Value
Sperm concentration (×10^6^/mL)	27.2 ± 32.7	27.5 ± 26.9	0.027
Number of spermatozoa (×10^6^/mL)	58.3 ± 64.1	63.5 ± 59.2	0.040
Progressive motility (%)	27.6 ± 17.2	34.6 ± 16.7	<0.0001
Total motile sperm count (×10^6^)	26.8 ± 35.3	33.1 ± 38.9	0.003
Sperm DNA Fragmentation ^1^ (%)	28.3 ± 25.1	16.3 ± 7.9	0.078

Data are expressed as mean ± SD unless otherwise stated; ^1^ paired data available in 14 patients.

**Table 3:** Embryological outcomes of T0 and T90 cycles.

	T0	T90	Unadjusted *p*-Value	Adjusted *p*-Value ^1^
Patient’s age at pick-up (years)	35.3 ± 3.2	35.4 ± 3.1	0.777	0.232
*N* retrieved oocytes	10.1 ± 3.6	9.7 ± 4.9	0.016	0.082
*N* MII oocytes	7.9 ± 2.9	7.9 ± 3.7	0.329	0.329
*N* inseminated oocytes	7.1 ± 2.2	7.2 ± 3.8	0.233	0.329
Fertilization rate (%)	39.8 ± 16.2	72.9 ± 19.9	<0.0001	<0.0001
Cleavage rate (%)	66.9 ± 24.7	71.5 ± 25.6	0.022	0.088
*N* cleavage-stage embryos	2.1 ± 1.1	3.3 ± 2.3	<0.0001	<0.0001
Top quality embryos (%)	64.8 ± 37.4	82.7 ± 27.6	<0.0001	<0.0001
Patients who developed at least one blastocyst (*N*, %)	4/77 (5%)	36/77 (47%)	<0.0001	<0.0001
*N* developed blastocysts	0.2 ± 0.6	1.1 ± 1.4	0.0001	0.001

Values are mean ± SD unless otherwise stated. ^1^ Alpha value adjusted by Hochberg stepwise test.

**Table 4:** Clinical outcomes of T0 and T90 cycles.

	T0	T90	Unadjusted *p*-Value	Adjusted *p*-Value ^1^
Cancelled embryo transfer (%)	9/77 (12)	3/77 (4)	0.068	0.614
Absence of fertilized oocytes (%)	4/77 (5)	0	0.048	0.476
Absence of viable embryos (%)	3/77 (4)	0	0.077	0.618
Delayed embryo transfer (freeze-all) (%)	2/77 (3)	3/77 (4)	0.736	0.974
*N* transferred embryos	1.8 ± 0.6	1.5 ± 0.6	0.022	0.240
Cycles with day 2–3 ET	65/68 (96%)	45/74 (61%)	<0.0001	<0.0001
Implantation (%)	2/124 (2%)	15/85 (18%)	<0.0001	<0.0001
Pregnancy (%)	2/65 (3%)	15/45 (33%)	0.0001	0.001
Live-birth rate (%)	0/124 (0%)	13/83 (16%) ^2^	<0.0001	<0.0001
Miscarriage (%)	2/2 (100%)	0/15 (0%)	<0.0001	<0.0001
Cycles with day 5 ET	3/68 (4%)	29/73 (40%)	<0.0001	<0.0001
Implantation (%)	0/4 (0%)	10/32 (31%)	0.475	0.974
Pregnancy (%)	0/3 (0%)	10/29 (34%)	0.577	0.974
Live-birth rate (%)	0/4 (0%)	10/32 (31%)	0.475	0.974
Miscarriage (%)	0%	0/10 (0%)	-	
*N* ET in freeze–thaw cycles	3	9		
Implantation (%)	1/4 (25%)	4/9 (44%)	0.974	0.974
Pregnancy (%)	1/3 (3%)	4/9 (44%)	0.551	0.974
Live-birth (%)	0/1 (0%)	-/9 ^3^	-	
Miscarriage (%)	1/1 (100%)	1/4 (25%)	0.819	0.974
Cumulative pregnancy rate per cycle (%)	1/71 (2%)	29/83 (35%)	<0.0001	
Cumulative live-birth rate per cycle (%)	0/132 (0%)	23/124 (19%) ^4^	<0.0001	<0.0001
Cumulative miscarriage (%)	3/3 (100%)	1/29 (3%)	0.0001	0.001
Cumulative pregnancy rate per couple (%)	1/77 (1%)	29/77 (38%)	<0.0001	<0.0001
Cumulative live-birth rate per couple (%)	0/77 (0%)	23/77 (30%) ^4^	<0.0001	<0.0001

ET, embryo transfer. ^1^ Alpha value adjusted by Hochberg stepwise test; ^2^ n. 2 ongoing clinical pregnancies; ^3^ n. 3 ongoing clinical pregnancies; ^4^ n. 5 ongoing clinical pregnancies.

**Table 5:** Neonatal characteristics of live births in T90 cycles.

	T90 Cycles
*N* live births	23
*N* lost follow-up	1
*N* ongoing pregnancies	5
Birthweight (grams)	3147.7 ± 321.5
*N* birthweight < 2500 g	0
Gestational age (weeks)	39.0 ± 1.1
*N* prematurity < 37 weeks	0
Birthweight centiles	34.7 ± 22.9
SDS-score	−0.4 ± 0.7

SDS, standard deviation score. Values are mean ± SD unless otherwise stated.

## Data Availability

The datasets analyzed during the current study are available from the corresponding author upon request.
